# Preclinical Evaluation of the SMNP-Adjuvanted R21 Malaria Vaccine Across Multiple Mouse Strains

**DOI:** 10.3390/vaccines14100861

**Published:** 2026-09-28

**Authors:** Gulbuse Turan, Kseniia Fedorova, Adrian V. S. Hill, Ahmed M. Salman

**Affiliations:** 1 Jenner Institute, University of Oxford, Oxford OX3 7DQ, UK; 2 Department of Bioengineering, Ege University, Izmir 35040, Turkey

**Keywords:** adjuvant, *Plasmodium falciparum*, pre-erythrocytic, SMNP, VLP vaccines

## Abstract

**Background**: Adjuvants can enhance the immunogenicity of virus-like particle (VLP) vaccines. VLP-based malaria vaccines (RTS,S and R21) have demonstrated the potential of this approach and have now been deployed in many countries. However, the efficacy of these vaccines decays over time and could be improved by better vaccine formulations. The development of next-generation saponin-based adjuvants, like SMNP, offers an opportunity to enhance immunogenicity and optimize antigen dose, potentially improving protective immunity. **Methods**: In this pre-clinical study, we evaluated the immunogenicity and efficacy of the newer, more protective R21 vaccine when formulated with 5 µg of SMNP adjuvant, tested at both a low (0.1 µg) and standard (1 µg) antigen (R21) dose. Three different mouse strains (*BALB/c*, *C57BL/6*, and *CD1*) were immunized using a two-dose regimen with a 3-week interval between doses. Naive mice served as controls. Three weeks post-boost, all animals were challenged with transgenic *Plasmodium berghei* parasites expressing *Plasmodium falciparum* circumsporozoite protein. We assessed vaccine-induced humoral responses and compared immunogenicity and protective efficacy across dose groups and strains relative to naive controls. **Results**: Across all strains, SMNP-adjuvanted R21 induced robust NANP-specific IgG responses, with both the low-dose and standard-dose groups generating high antibody titers. Patterns of immunogenicity varied among mouse strains, but the low-dose R21 + SMNP group consistently elicited responses comparable to the standard-dose group. Although both vaccine doses delayed the onset of blood-stage parasitemia in all mouse strains, the low-dose groups showed a trend toward reduced efficacy compared with the standard dose, but this difference was not statistically significant. **Conclusions**: Our findings demonstrate that R21 formulated with SMNP is highly immunogenic, and that substantial dose-sparing may be achievable without compromising immune responses. This work supports further exploration of SMNP-adjuvanted VLP vaccines and highlights the potential for dose-optimized strategies to improve the accessibility and scalability of next-generation malaria vaccines.

## 1. Introduction

The immunogenicity of subunit vaccines is partially determined by the adjuvant system with which the antigen is formulated [1]. Recombinant protein antigens are generally unable to generate strong and durable immunity on their own because they lack the inflammatory and innate immune signals required to efficiently activate antigen-presenting cells and prime adaptive responses [2]. For this reason, adjuvants are essential components of protein-based vaccine platforms, where they function not only as immune enhancers but also as determinants of response quality. By influencing antigen uptake, innate immune activation, lymph node trafficking, T-cell polarization, B-cell maturation, and memory formation, adjuvants can substantially alter the magnitude, durability, and functional profile of vaccine-induced protection [2,3,4].

Adjuvants used in malaria vaccine development have differed widely in composition and mechanism. Aluminum-based formulations have historically been favored because of their safety and ability to enhance antibody responses, but they commonly promote Th2-skewed immunity and may be insufficient when broader cellular and functional antibody responses are required [5,6,7]. More complex systems, such as AS01, combine innate immune receptor stimulation with particulate delivery, thereby enhancing both humoral and T-cell-mediated immunity [8,9]. Similarly, Matrix-M has demonstrated the value of saponin-based nanoparticles in malaria vaccination by promoting antigen drainage to local lymph nodes, inducing strong antibody responses, and supporting balanced Th1/Th2 CD4^+^ T-cell immunity [10,11,12]. Current literature suggests that particulate adjuvants capable of coordinating antigen delivery with innate immune activation may be especially suitable for improving circumsporozoite protein (CSP)-based vaccine responses.

Saponin-containing adjuvants are of particular interest because they can enhance both antibody and cellular immunity [13,14]. QS-21, one of the most potent saponin-derived immunostimulants, has been incorporated into several modern adjuvant systems, including AS01 and Matrix-M-related formulations [2,14]. Although free QS-21 can cause hemolytic activity and reactogenicity, these effects can be reduced when saponins are incorporated into liposomal or nanoparticle structures. This has supported the development of saponin-based particulate adjuvants that retain strong immunostimulatory activity while improving tolerability. SMNP (Saponin/MPLA Nanoparticle) is a newer ISCOM (immunostimulating complex)-like adjuvant platform that combines saponins with monophosphoryl lipid A (MPLA), a Toll-like receptor 4 (TLR4) agonist [15,16]. Through this design, SMNP integrates two complementary immunological features: particulate saponin-mediated antigen delivery and innate immune activation through TLR4 signaling [15]. Preclinical studies have shown that SMNP can enhance lymphatic flow, increase lymph node permeability, and stimulate dendritic cell cytokine production, all of which are processes associated with efficient vaccine priming. In non-human primates, SMNP has also produced stronger humoral responses than aluminum hydroxide, supporting its potential as a potent adjuvant platform for recombinant protein vaccines [17]. It has recently been produced as GMP-grade and has entered phase 1 clinical trials (NCT06033209) [18].

Previous studies in murine transgenic *Plasmodium berghei* challenge models have shown that R21 formulated with saponin-based ISCOM adjuvants, including Abisco-100 or Matrix-M, can induce high levels of sterile protection, whereas R21 administered without adjuvant does not provide comparable protection [19,20]. Building on this evidence, the present study was designed to evaluate the immunogenicity and protective efficacy of R21 formulated with the saponin/MPLA nanoparticle adjuvant SMNP, rather than to re-establish the requirement for adjuvantation using an R21-alone control. We further assessed whether a ten-fold reduction in R21 antigen dose could retain immunogenicity and protection and whether responses to R21/SMNP were consistent across BALB/c, C57BL/6, and CD1 mouse strains. Although the absence of a contemporaneous unadjuvanted R21 group precludes direct quantification of the incremental contribution of SMNP relative to R21 alone in the present study, this design enabled focused evaluation of the R21/SMNP formulation and its dose-sparing potential.

Low and standard doses of R21 formulated with SMNP were tested first in *BALB/c* mice, which are widely used in pre-erythrocytic malaria models due to their well-characterized immune responses, then followed by *C57BL/6* and *CD1* strains. Importantly, differences in vaccine efficacy between mouse strains may also be influenced by major histocompatibility complex (MHC) restriction, which determines how vaccine-derived antigens are processed and presented to T-cells. Each inbred mouse strain expresses distinct MHC haplotypes (e.g., H-2d in *BALB/c* and H-2b in *C57BL/6*), resulting in variability in epitope presentation and subsequent T-cell activation [21]. As a consequence, even when exposed to the same antigen, different mouse strains may generate divergent immune responses due to their MHC-specific peptide recognition. These immunogenetic differences can impact both the magnitude and quality of the immune response and, hence, the efficacy elicited by a vaccine. To account for these biological variations, two inbred mouse strains (*BALB/c* and *C57BL/6*) and one outbred strain (*CD1*) were utilized in this study. The inclusion of *CD1* allowed for an expanded evaluation of immune responses in a genetically diverse background, as *CD1* has a range of MHC haplotypes, providing insights into how genetic variability influences vaccine-induced immunity. By extending the analysis to an outbred strain, this approach aimed to enhance the translational relevance of the findings and assess the robustness of immune responses across different genetic profiles, mimicking the massive variety within the human population.

## 2. Materials and Methods

### 2.1. Adjuvants

Saponin/MPLA nanoparticles (SMNPs) are ISCOM-like particulate adjuvants composed of saponin, cholesterol, dipalmitoylphosphatidylcholine (DPPC), and monophosphoryl lipid A (MPLA; PHAD^®^). They were prepared by co-assembly of these components at a molar ratio of 10:10:2.5:1 (saponin:cholesterol:DPPC:MPLA), yielding homogeneous nanocage-like particles of approximately 40 nm in diameter. MPLA is physically incorporated within the nanoparticle structure, rather than admixed with preformed saponin particles, and retained TLR4-stimulatory activity following formulation.

SMNPs were supplied by the Massachusetts Institute of Technology (MIT) under a material transfer agreement for use in the vaccination experiments and were received as a pre-formulated material. The adjuvants were stored and handled according to the supplier’s instructions and mixed with R21 immediately before immunization. The SMNP’s composition and physicochemical/functional characterization have been described previously [15].

### 2.2. Animals

Animal procedures were performed under the UK Animals (Scientific Procedures) Act 1986 following approval by the University of Oxford Animal Care and Ethical Review Committee. Procedures were designed and conducted in accordance with the principles of replacement, reduction, and refinement. Animals were maintained under specific pathogen-free conditions in individually ventilated cages with constant temperature and humidity and a 12-h light/dark cycle. Where short-term anesthesia was required, animals received either intramuscular xylazine (~7 mg/kg) and ketamine (~70 mg/kg) or inhaled IsoFlo^®^ at 3.5% with oxygen supplied at 2 L/minute. Animals were monitored throughout the experiments and euthanized at the experimental endpoint by an approved Schedule 1 method.

### 2.3. Immunization

Female *BALB/c*, *C57BL/6*, and *CD1* mice, 6 weeks of age, were obtained from Inotiv (formerly Envigo). Mice were immunized under isoflurane anesthesia by intramuscular administration of 50 mcl vaccine into one hind limb. Each vaccine dose contained either 0.1 or 1 µg of VLP formulated with 5 µg of SMNP. Sterile phosphate-buffered saline (PBS) was added as required to achieve the final injection volume. The 5 µg of SMNP dose contained 5 μg of saponin and 0.5 μg of MPLA and was selected based on previous preclinical studies in which this dose was used in HIV and SARS-CoV-2 vaccine models [15,22]. A prime–boost–challenge schedule was used, with approximately three weeks between each step. Prepared vaccines were kept on ice after preparation and administered within 1 h. Blood samples were collected two days before booster immunization and again before parasite challenge. Samples were allowed to clot overnight at 4 °C, centrifuged at 13,000 rpm for 10 min, and sera were stored at −20 °C until use.

### 2.4. Parasite Challenge

The mosquito infection, sporozoite preparation, and challenge procedures followed the approach described previously [23], with the chimeric parasite line PfCSP(r)PbCSP used in the present study. Briefly, frozen parasitized red blood cells were thawed, and 100–300 mcl of infected blood were administered intraperitoneally to a naïve *BALB/c* mouse. Parasitemia was assessed five to six days later using Giemsa-stained thin blood films. Anesthetized infected mice were then exposed for 10–15 min to starved female *Anopheles stephensi* mosquitoes aged four to seven days old. Following feeding, mosquitoes were maintained at 19–23 °C under humidified conditions and provided with fructose solution. Salivary glands were dissected 21–23 days after feeding, and sporozoites were released by homogenization in cell-culture medium on ice and quantified using a hemocytometer. Approximately three weeks after booster immunization, vaccinated and naïve mice received 1000 sporozoites intravenously via the lateral tail vein. Blood-stage infection was monitored from day five after the challenge using thin blood films. Animals with parasite-positive smears on three consecutive days were euthanized. For infected mice, three parasitemia measurements were used to calculate the time to 1% parasitemia by linear regression. Animals remaining blood-smear negative through day 14 after challenge were classified as protected.

### 2.5. Thin Blood Films

Thin blood films and calculation of time to 1% parasitemia were performed as described previously [23,24]. Briefly, blood obtained from the tail snip was spread onto glass slides, air-dried, methanol-fixed, and stained with 5% Giemsa for 0.5–1 h. Slides were examined using light microscopy with a 100× oil immersion-objective. For infected animals, percentage parasitemia values were log-10 transformed and plotted against time after challenge. Linear regression was applied to the linear phase of the blood-stage growth curve using GraphPad Prism 10 to calculate the time at which 1% parasitemia was reached.

### 2.6. Anti-NANP IgG ELISA

Nunc-Immuno Maxisorp 96-well plates (Thermo Fisher Scientific, 442404, Roskilde, Denmark) were coated overnight with 2 µg/mL of (NANP)6C peptide in carbonate-bicarbonate coating buffer (Sigma-Aldrich, C3041). Plates were washed four times with PBS-Tween^®^ 20 (Sigma-Aldrich, P7949, Saint Louis, MO, USA) (0.05% *v*/*v*) and blocked with Casein in PBS (1% (*w*/*v*) solution of purified casein) (Thermo Scientific, 37528) for 1 h at room temperature. Post-prime samples were diluted 1 in 1000, whereas post-boost samples were diluted 1 in 10,000. Samples were tested in triplicate. Anti-NANP monoclonal antibody 2A10 (Absolute Antibody, Ab03231-3.0) was used to generate a standard curve with 10-point two-fold serial dilutions. Following a 2-h incubation at room temperature, plates were washed four times with PBS-T and incubated for 1 h with alkaline phosphatase-conjugated anti-mouse whole IgG (1 in 1000, Sigma-Aldrich, A3562). Signal was developed using p-nitrophenyl phosphate (Sigma, N2765) at 1 mg/mL in diethanolamine buffer (Sigma-Aldrich, 34064) and measured as optical density (OD) at 405 nm using a BioTek ELx808 microplate reader (BioTek Instruments, Inc., Winooski, VT, USA). ELISA units were calculated from the linear range of the standard curve. Samples with OD values < 0.1 were assigned a titer of 100 as the lowest quantifiable value represented by the standard curve and classified as non-responders.

### 2.7. IgG Subclass ELISA

NANP-specific IgG subclasses were measured using Maxisorp 96-well plates coated overnight at 4 °C with 2 µg/mL (NANP)6C peptide in carbonate–bicarbonate coating buffer. Plates were washed with PBS-Tween and blocked with Casein in PBS for 2 h at room temperature. Serum samples were normalized according to their total anti-NANP IgG response to produce an OD405 value of approximately 1. The resulting sample-specific dilution was subsequently used for all IgG subclass measurements from that serum sample, and each sample was tested in duplicate in Casein in PBS. A reference serum pool was included on each plate as an internal control. After incubation for 2 h at 37 °C, plates were washed and incubated for 1 h at 37 °C with alkaline phosphatase-conjugated antibodies specific for mouse IgG1 (1 in 4000, Southern Biotech, Birmingham, AL, USA), IgG2a (1 in 4000, Southern Biotech), IgG2b (1 in 4000, Southern Biotech), IgG2c (1 in 4000, Southern Biotech), or IgG3 (1 in 1000, Abcam, Singapore). Plates were developed with p-nitrophenylphosphatase at 1 mg/mL in diethanolamine buffer and read at 405 nm. IgG subclass responses are reported as optical density values.

### 2.8. Avidity ELISA

Anti-NANP antibody avidity was assessed using the previously described NaSCN-displacement ELISA [23]. Serum samples were diluted to the concentration producing an OD405 of approximately 1 in the standard anti-NANP ELISA and added in duplicate to plates coated with 2 µg/mL (NANP)6C peptide. After incubation for 2 h at RT and washing six times with PBS-Tween, samples were exposed for 15 min to NaSCN concentrations of 0, 0.5, 1, 1.5, 2, 2.5, 3, and 3.5 M. Plates were washed and incubated for 1 h with alkaline phosphatase-conjugated goat anti-mouse whole IgG. Bound antibody was detected using p-nitrophenyl phosphate at 1 mg/mL in diethanolamine buffer, with absorbance measured at 405 nm. Avidity was expressed as the NaSCN IC50, defined as the concentration required to reduce ELISA signal to 50% of that observed in the absence of NaSCN.

### 2.9. Statistical Analysis

Statistical analyses were performed using GraphPad Prism version 10 (GraphPad, Boston, MA, USA). Power calculations were based on antibody magnitude (one-way ANOVA) and time to 1% parasitemia. With 6–8 mice per group, the study provided ≥80% power to detect large effect sizes of f ≥ 0.4 for antibody responses and a hazard ratio ≥ 3 for time to parasitemia. Correlation analyses were designed to detect associations of ρ ≥ 0.6. Comparisons between two independent groups were performed using the Mann–Whitney test. Paired observations were analyzed using the Wilcoxon matched-pairs test or the Friedman test, as appropriate. Comparisons involving more than two non-parametric groups were performed using the Kruskal–Wallis test followed by Dunn’s multiple comparison test. Spearman’s rank correlation was used to assess associations between variables. Challenge outcomes were visualized using Kaplan–Meier curves and compared by the log-rank (Mantel–Cox) test. Statistical significance was defined as *p* < 0.05, with significance indicated as * *p* < 0.05, ** *p* < 0.01, or *** *p* < 0.001.

## 3. Results

To assess the immune response variability influenced by antigen dose and genetic background, two doses of R21 (0.1 µg and 1 µg) were tested with SMNP at a dose of 5 µg. Both formulations were evaluated across three mouse strains: BALB/c (*n* = 6), C57BL/6 (*n* = 6), and CD1 (*n* = 7–8).

### 3.1. Low- and Standard-Dose R21 Formulated with SMNP Is Immunogenic and Partially Protective in BALB/c Mice

Two doses of vaccination were administered, and serum was collected at two time points: post-prime and post-boost (*n* = 6). (Figure 1A). Vaccinated groups included low-dose and standard-dose R21 combined with SMNP adjuvant, whereas a naïve group served as a negative control (Figure 1B). For challenge, 1000 transgenic sporozoites from the PfCSP(r)PbCSP parasite line were injected intravenously. All mice were monitored from day 5 post-challenge via thin blood smears. IgG immune response against NANP repeats was assessed in both prime and boost serum samples using ELISA (Figure 1C). *BALB/c* mice vaccinated with the low-dose R21 group displayed a significant immune response compared to naïve samples with a *p*-value of 0.0059. The standard dose of R21 produced a significant amount of IgG after the prime dose (*p* = 0.0059). All groups showed a statistically higher immune response than naïve samples after the boost vaccination (low-dose group, *p* = 0.0041; standard-dose group, *p* = 0.0084). There was no significant difference between the vaccinated groups after either prime or boost vaccination. The fold change in anti-NANP IgG antibody levels between the prime and boost immunizations was also analyzed, but no significant differences were observed between the groups (Supplementary Figure S1A).

To assess the quality of the antibody response, the chaotropic reagent NaSCN was used (Figure 1D). The half-maximal inhibitory concentration (IC50) of NaSCN is displayed as molarity (M) for post-boost NANP-specific IgG titers. The distribution of sample avidity was found to be wider (between 0.345 and 1.355 M) in the low-dose group. The median of the low-dose group was higher than the standard-dose group, although the difference was not statistically significant (*p* = 0.4848). The association between antibody avidity and time to reach 1% parasitemia was also assessed in *BALB/c* mice. No significant correlation was observed between these variables (Supplementary Figure S2A).

The standard-dose R21 + SMNP demonstrated the highest efficacy, providing 66.7% protection by preventing the onset of parasitemia in four of six subjects (*p* = 0.0005) and provided delayed infection compared with the naïve group (*p* = 0.0344) (Figure 1E). The low-dose R21 + SMNP formulation achieved 50% efficacy (*p* = 0.0005) and also delayed time to 1% parasitemia in infected mice (*p* = 0.0110). Both groups were compared with each other using the Mantel–Cox test, and no significance was detected (*p* = 0.7206).

IgG subclass distribution (IgG1, IgG2a, IgG2b, and IgG3) was assessed and displayed as optical density (OD) at 405 nm (Figure 1F). OD values remained below 0.25 for all subclasses in *BALB/c* mice. Samples from the standard-dose group showed a greater tendency towards IgG1 responses compared to G2b and G3 (*p* = 0.0048, 0.0437, respectively). In the low-dose group, IgG2a and IgG1 appeared to be more readily induced; however, given the low OD values observed, these differences were not statistically significant. In all subclasses, low-dose and standards-dose groups produced statistically higher OD values compared with naïve groups (IgG1, *p* = 0.0229, 0.0001; IgG2a, *p* = 0.0005, 0.0078; IgG2b, *p* = 0.0044, 0.0010; IgG3, *p* = 0.0014, 0.0031, respectively).

To evaluate the relationship between antibody responses and protection, post-boost NANP-specific IgG titers from all groups were combined and plotted against the time to %1 parasitemia (Figure 1G). Spearman’s correlation analysis showed a strong positive correlation (r = 0.7580, *p* = 0.0003). Linear regression analysis yielded an R^2^ value of 0.1888, which indicates that approximately 18% of the variation in time to 1% parasitemia could be explained by NANP-specific IgG levels, with higher antibody titers being associated with delayed onset of parasitemia and improved protection.

### 3.2. Low (0.1 µg)- and Standard (1 µg)-Dose R21 Formulated with SMNP Is Immunogenic but Not Protective in C57BL/6 Mice

Two doses of vaccination were administered, and serum was collected at two time points: post-prime and post-boost (*n* = 6) (Figure 2A). Vaccinated groups included low-dose and standard-dose R21 combined with SMNP adjuvant, whereas a naïve group served as a negative control (Figure 2B). For challenge, 1000 transgenic sporozoites from the PfCSP(r)PbCSP parasite line were injected intravenously. All mice were monitored from day 5 post-challenge via thin blood smears. In *C57BL/6*, prime vaccination elicited significantly higher NANP-specific IgG immune response in low-dose and standard-dose groups compared to naïve samples (*p* = 0.0414, 0.0006, respectively) (Figure 2C). After boost, low-dose and standard-dose groups produced a higher amount of IgG in each subject (*p* = 0.0278, 0.0073, respectively). Compared to the naïve group, NANP-specific IgG titers induced by post-boost vaccination were significantly higher in both groups (*p* = 0.0479, 0.0004, respectively). There was no statistically significant difference between the vaccinated groups after either vaccination. The fold change in anti-NANP IgG antibody levels from the prime to the boost immunization was also analyzed and no significant differences were found between groups (Supplementary Figure S1B).

Avidity of NANP-specific IgG titers was assessed via increasing NaSCN molarity (Figure 2D). The half-maximal inhibitory concentration (IC50) of NaSCN is displayed as molarity (M) for post-boost NANP-specific IgG titers. The distribution of sample avidity was found to be wider (between 0.104 and 1.108 M) in the low-dose group. The median of the low-dose group was higher than the standard-dose group, although the difference was not statistically significant (*p* = 0.0931). The association between antibody avidity and time to reach 1% parasitemia was also assessed in *C57BL/6* mice. No significant relationship was observed (Supplementary Figure S2B).

Although both low- and standard-dose vaccinated groups demonstrated a significant delay to 1% parasitemia compared with naïve mice (*p* = 0.0013, 0.0034, respectively), only the standard-dose R21 formulation provided very low protection, preventing parasitemia in one mouse out of six (16.7%) (*p* = 0.0005) (Figure 2E). The low-dose R21 group failed to confer any protection, as all mice in this group eventually became infected (*p* = 0.0005). Comparison of the two vaccinated groups using the Mantel–Cox test revealed no significant difference between them (*p* = 0.8210).

NANP-specific IgG subclass distribution (IgG1, IgG2b, IgG2c, and IgG3) was assessed via ELISA and displayed as OD at 405 nm (Figure 2F). Among the standard-dose samples, IgG2c exhibited the highest OD values, although the maximum OD for all subclasses remained below 0.5. In the low-dose group, IgG1 and IgG2c appeared to be more readily induced; however, differences among subclasses were not statistically significant. Compared with the naïve group, the low-dose group showed significantly higher OD values for IgG1, 2b, and 2c (*p* = 0.0125, 0.0386, and 0.0331, respectively). In contrast, a significantly higher IgG3 response was only observed in the standard-dose group (*p* = 0.0125).

Post-boost NANP-specific IgG titers from all groups were combined and plotted against the time to %1 parasitemia to evaluate the relationship between antibody responses and protection (Figure 2G). Linear regression analysis yielded an R^2^ value of 0.1565, which indicates that only approximately 15% of the variation in time to 1% parasitemia could be explained by anti-NANP IgG levels.

### 3.3. Low (0.1 µg)- and Standard (1 µg)-Dose R21 Formulated with SMNP Is Immunogenic and Partially Protective in CD1 Mice

Two doses of vaccination were administered, and serum was collected at two time points: post-prime and post-boost (*n* = 8, *n* = 7 for naïve group) (Figure 3A). Vaccinated groups included low-dose and standard-dose R21 combined with SMNP adjuvant, whereas a naïve group served as a negative control (Figure 3B). For challenge, 1000 transgenic sporozoites from the PfCSP(r)PbCSP parasite line were injected intravenously. All mice were monitored from day 5 post-challenge via thin blood smears. After the prime vaccination in *CD1*, low- and standard-dose R21 + SMNP elicited a significant immune response against NANP compared to naïve samples (*p* = 0.0050, 0.0002) (Figure 3C). After the booster dose, a significantly higher NANP-specific IgG was detected in both the low- and standard-dose groups compared with the naïve control (*p* = 0.0021, 0.0012, respectively). There was no statistically significant difference between the vaccinated groups after either vaccination (*p* > 0.9999). Following the boost, both groups produced a higher amount of NANP-specific IgG compared to prime samples (*p* = 0.0003, 0.0011, respectively). Fold change in antibody levels from the prime to the boost immunization was analyzed. No significant differences were found between groups in fold change of IgG responses (Supplementary Figure S1C).

Avidity of NANP-specific IgG titers was assessed via increasing NaSCN molarity (Figure 3D). The half-maximal inhibitory concentration (IC50) of NaSCN is displayed as molarity (M) for post-boost NANP-specific IgG titers. The distribution of sample avidity was found to be wider (between 0.363 and 1.427 M) in the standard-dose group. The median of the low-dose group was similar to that of the standard-dose group; hence, there was no statistically significant difference found (*p* = 0.8182). The association between antibody avidity and time to reach 1% parasitemia was also assessed in *CD1* mice. Linear regression analysis yielded an R2 value of 0.4219, which indicates that approximately 42% of the variation in time to 1% parasitemia could be explained by the avidity of anti-NANP IgG levels observed. Spearman’s correlation analysis showed a significant positive correlation between avidity levels and protection (r = 0.6082, *p* = 0.0408) (Supplementary Figure S2C).

Low-dose and standard-dose formulations successfully delayed the infection to different levels (*p* = 0.0024, 0.0073, respectively) (Figure 3E). The R21 standard-dose combination with SMNP demonstrated the highest efficacy, achieving 62.5% protection by preventing the onset of parasitemia in five out of eight subjects (*p* < 0.0001). Efficacy was 50% for low-dose R21 combined with SMNP (*p* < 0.0001). Both groups were compared with each other individually using the same test, and no significance was detected (*p* = 0.6002).

Post-boost NANP-specific IgG1, 2a, 2b, and 3 responses were assessed via ELISA and displayed as OD at 405 nm (Figure 3F). In all subclasses, low-dose and standard-dose groups produced statistically higher OD values compared with naïve groups (IgG1, *p* = 0.0229, 0.0001; IgG2a, *p* = 0.0005, 0.0078; IgG2b, *p* = 0.0044, 0.0010; IgG3, *p* = 0.0014, 0.0031, respectively). Among the standard-dose samples, IgG2a exhibited the lowest OD value, with the maximum OD value remaining below 0.4. In the low-dose group, IgG1 and IgG3 appeared to be more readily induced; however, differences among subclasses were not statistically significant (*p* > 0.9999). Overall, there was no difference in NANP-specific IgG subclasses between vaccinated groups.

Post-boost NANP-specific IgG titers from all groups were combined and plotted against the time to %1 parasitemia to evaluate the relationship between antibody responses and protection (Figure 3G). Spearman’s correlation analysis showed a significant positive correlation between antibody titer and protection (r = 0.8111, *p* < 0.0002). Linear regression analysis yielded an R2 value of 0.3635, which indicates that approximately 36% of the variation in time to 1% parasitemia could be explained by anti-NANP IgG levels.

## 4. Discussion

Our study investigated the capacity of SMNP to trigger R21-induced immunity and protection for the first time in the literature. We tested both standard (1 µg)- and low (0.1 µg)-dose R21 across *BALB/c*, *C57BL/6*, and *CD1* mice to assess whether SMNP could help induce robust antibody response, delay or prevent parasitemia, and maintain activity across distinct immunological backgrounds. Across all strains, SMNP-adjuvanted R21 induced robust NANP-specific IgG responses, with both the low-dose and standard-dose groups generating high antibody titers. Patterns of immunogenicity varied among mouse strains, but the low-dose R21 + SMNP group consistently elicited humoral immune responses comparable to the standard-dose group. Although both vaccine doses delayed the onset of blood-stage parasitemia in all mouse strains, the low-dose groups showed a trend toward reduced efficacy compared with the standard dose, but this difference was not statistically significant.

The differences in protection between mouse strains further support a role for host immunological background in determining vaccine outcome. *BALB/c* mice generated strong NANP-specific IgG responses and demonstrated the most favorable protective profile, including substantial delays in parasitemia and partial protection following both vaccine formulations. As antibodies targeting the CSP NANP repeat region are closely associated with protection, the robust NANP-specific antibody responses observed in this strain may have contributed to its improved outcome [25].

In contrast, *C57BL/6* mice demonstrated lower levels of protection despite the induction of NANP-specific IgG responses. BALB/c and C57BL/6 mice are often described as having relatively Th2- and Th1-biased immune backgrounds, respectively [26]. However, cellular immune responses and Th1/Th2-associated immune markers were not evaluated in the present study. Therefore, the observed differences in protection cannot be attributed directly to differential Th1/Th2 polarization. More broadly, strain-specific variation may reflect multiple immunological and genetic factors, including MHC haplotype, antigen presentation, T-cell help, germinal center activity, antibody affinity maturation, immune-memory development, and other host-dependent determinants of vaccine responsiveness. Therefore, the underlying mechanism of strain-dependent differences in vaccine-induced protection remains unresolved.

Unlike inbred strains, *CD1* showed a more heterogenous NANP-specific antibody response. Inter-individual variability was most evident in the dispersion of individual NANP-specific IgG titers and the range of time to 1% parasitemia within each vaccine group. This was particularly relevant in *CD1* mice, where the outbred genetic background may contribute to greater heterogeneity in antibody magnitude and protective outcome compared with inbred *BALB/c* and *C57BL/6* mice.

The relationship between NANP-specific IgG titers and protection provides further insight into the contribution of humoral immunity. Across vaccinated animals, higher NANP-specific IgG titers were significantly associated with a longer time to 1% parasitemia, which supports the previous evidence that anti-repeat antibodies are an important component of protection induced by R21 and other anti-CSP repeat-based vaccines. However, the relatively low proportion of variance explained by the linear regression indicates that antibody concentration alone was insufficient to account for differences in the timing of parasitemia. This finding suggests that qualitative characteristics of the antibody response may be important determinants of protection. Fc-receptor engagement, complement activity, and functional inhibition of sporozoite invasion may therefore provide additional information. Although our findings indicate that substantial immune responses can be generated with reduced antigen input, larger studies incorporating durability and longer-term protection will be required to determine the extent to which antigen dose can be reduced without compromising the quality or persistence of vaccine-induced immunity.

Our study has several limitations. Although previous work has shown that unadjuvanted R21 is non-protective in comparable *P. berghei* transgenic challenge models, the absence of an unadjuvanted R21 control group means that the present experiments cannot quantify the specific contribution of SMNP relative to R21 alone [20]. The relatively small group sizes also limit statistical power. In addition, differences in intrinsic susceptibility to the *P. berghei* among BALB/c, C57BL/6, and CD1 mice may have contributed to the observed variation in protective efficacy. As the study was designed only to measure humoral immune response via antibodies and cellular response was not directly measured, the mechanisms underlying these strain-dependent differences remain unresolved. Finally, only a single SMNP dose was evaluated, which precludes conclusions regarding the optimal adjuvant dose.

Despite these limitations, our findings provide the first evidence of robust humoral immunity of R21 formulated with SMNP and its protection in preclinical malaria models. They also open important questions regarding how SMNP shapes cellular immunity and immune memory, and whether these mechanisms are conserved across different vaccine antigens and host backgrounds. Further investigation of these parameters will be important for determining the broader utility of SMNP in R21 and other VLP-based malaria vaccine formulations.

## Figures and Tables

**Figure 1:**
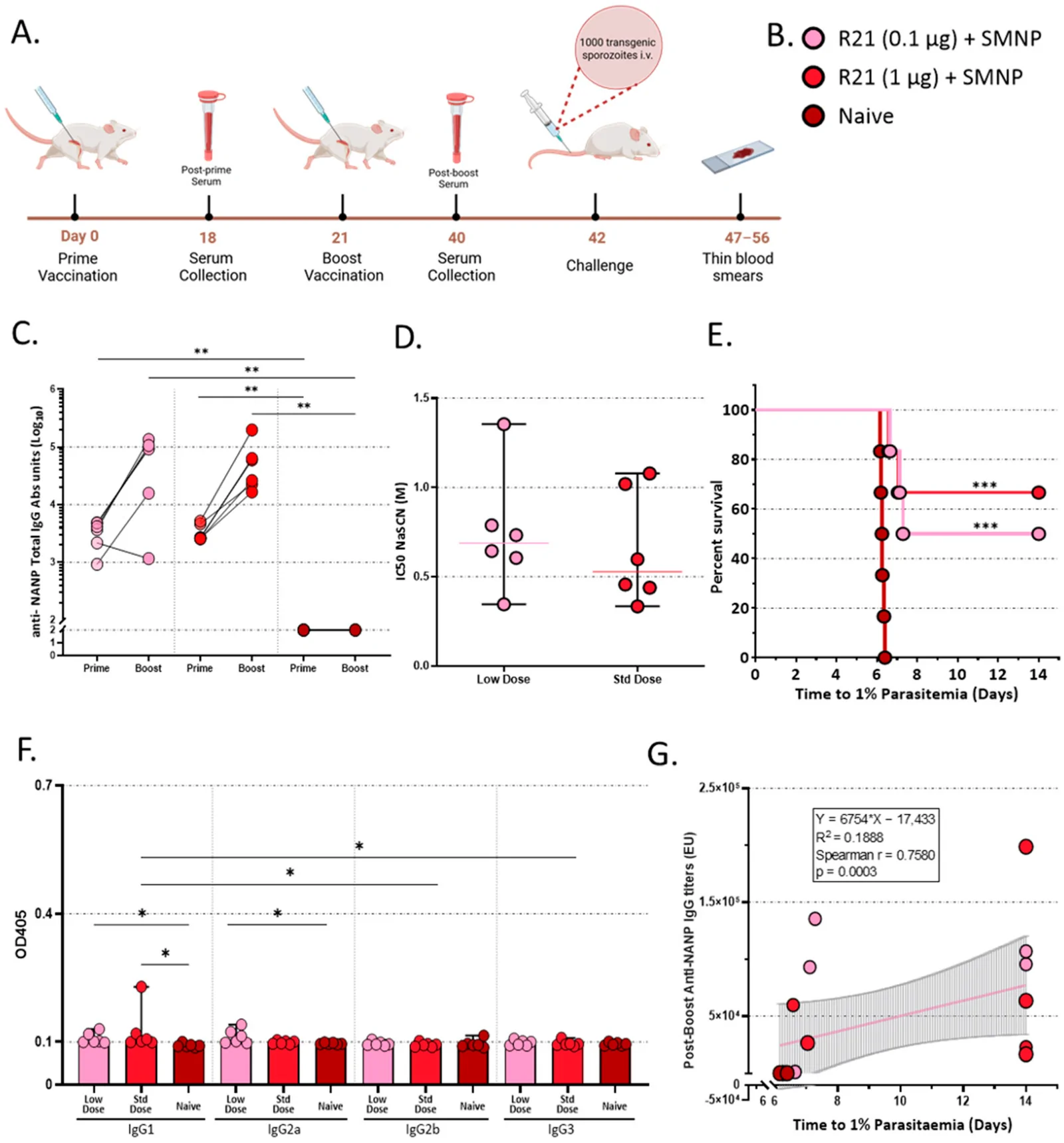
Low- and standard-dose R21 formulated with SMNP confer protection in *BALB/c* mice. (**A**) Experimental design. Here, 6-week-old female *BALB/c* mice (*n* = 6 per group) received two intramuscular immunizations, three weeks apart, containing either 0.1 µg or 1 µg R21 formulated with 5 µg of SMNP. Blood was collected via tail bleeding 2–3 days before boost vaccination and again before challenge. Approximately three weeks after boosting, mice were challenged intravenously with 1000 transgenic sporozoites. (**B**) Vaccination groups included in the study. (**C**) Anti-NANP IgG responses measured following prime and booster immunization. Samples yielding an OD below 0.1 were assigned a titer of 100 and classified as non-responders; these values are shown at the dotted threshold. Post-boost responses were compared with the naïve group using the Kruskal–Wallis test followed by Dunn’s multiple comparisons. (**D**) Avidity of NANP-specific IgG following booster immunization. Data shown as median with 95% confidence interval. Groups were compared using the Mann–Whitney test. (**E**) Protection following sporozoite challenge, shown as Kaplan–Meier curves. Differences between groups were evaluated using the log-rank (Mantel–Cox) test. (**F**) NANP-specific IgG subclass responses, shown as OD405 values for IgG1, IgG2a, IgG2b, and IgG3. For comparisons among independent groups, we used a Kruskal–Wallis test followed by Dunn’s multiple-comparisons test. Paired samples were analyzed using a Friedman test followed by Dunn’s multiple-comparisons test. (**G**) Relationship between post-boost anti-NANP IgG titers and time to 1% parasitemia. Data from all experimental groups were combined for this analysis. Mice remaining parasite-negative until the end of the study were assigned a value of 14 days. The fitted regression line and its 95% confidence interval are shown, together with the corresponding R^2^ value. Association between antibody titer and time to parasitemia was assessed using Spearman’s rank correlation. Statistical significance was defined as *p* ≤ 0.05 and displayed with asterisks (* = *p* ≤ 0.05, ** = *p* ≤ 0.01 and *** = *p* ≤ 0.001).

**Figure 2:**
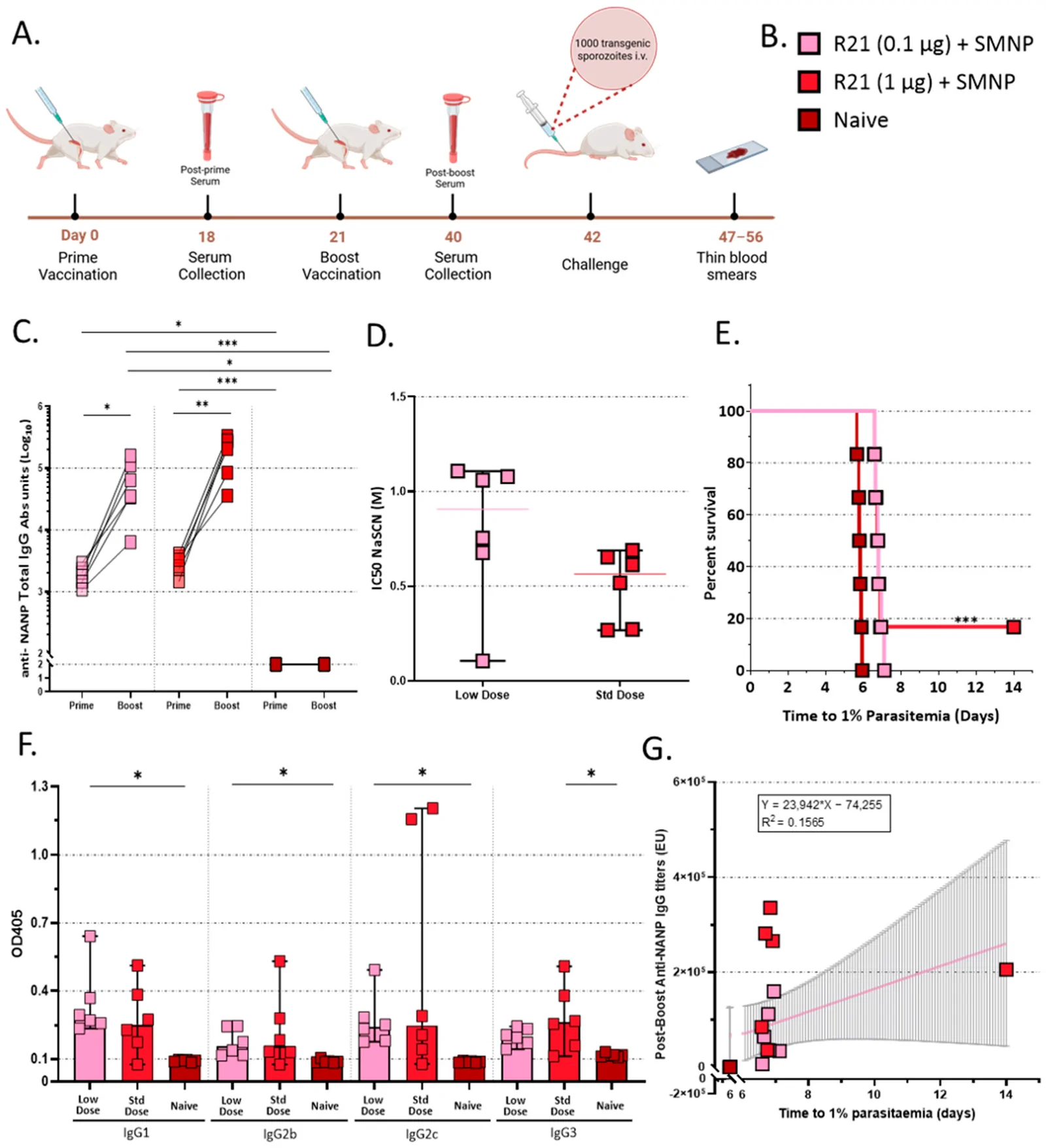
Low dose and standard dose of R21 formulated with SMNP do not confer protection in *C57BL/6* mice. (**A**) Experimental design. Here, 6-week-old female *C57BL/6* mice (*n* = 6 per group) received two intramuscular immunization, three weeks apart, containing either 0.1 µg or 1 µg of R21 formulated with 5 µg of SMNP. Blood was collected via tail bleeding 2–3 days before boost vaccination and again before challenge. Approximately three weeks after boosting, mice were challenged intravenously with 1000 transgenic sporozoites. (**B**) Vaccination groups included in the study. (**C**) Anti-NANP IgG responses measured following prime and booster immunization. Samples yielding an OD below 0.1 were assigned a titer of 100 and classified as non-responders; these values are shown at the dotted threshold. Statistical analysis was performed using the Kruskal–Wallis test followed by Dunn’s multiple comparisons. (**D**) Avidity of NANP-specific IgG following booster immunization. Data are shown as a median with a 95% confidence interval. Groups were compared using the Mann–Whitney test. (**E**) Protection following sporozoite challenge, shown as Kaplan–Meier curves. Differences between groups were evaluated using the log-rank (Mantel–Cox) test. (**F**) NANP-specific IgG subclass responses, shown as OD405 values for IgG1, IgG2a, IgG2b, and IgG3. For comparisons among independent groups, a Kruskal–Wallis test followed by Dunn’s multiple-comparisons test was used. Paired samples were analyzed using a Friedman test followed by Dunn’s multiple-comparisons test. (**G**) Exploratory simple linear regression was used to visualize the relationship between post-boost anti-NANP IgG titers and time to 1% parasitemia. Data from all experimental groups were combined for this analysis. Mice remaining parasite-negative until the end of the study were assigned a value of 14 days. The fitted regression line and its 95% confidence interval are shown, together with the corresponding R^2^ value. Association between antibody titer and time to parasitemia was not assessed as only one mouse remained parasitemia-free. Statistical significance was defined as *p* ≤ 0.05 and displayed with asterisks (* = *p* ≤ 0.05, ** = *p* ≤ 0.01 and *** = *p* ≤ 0.001).

**Figure 3:**
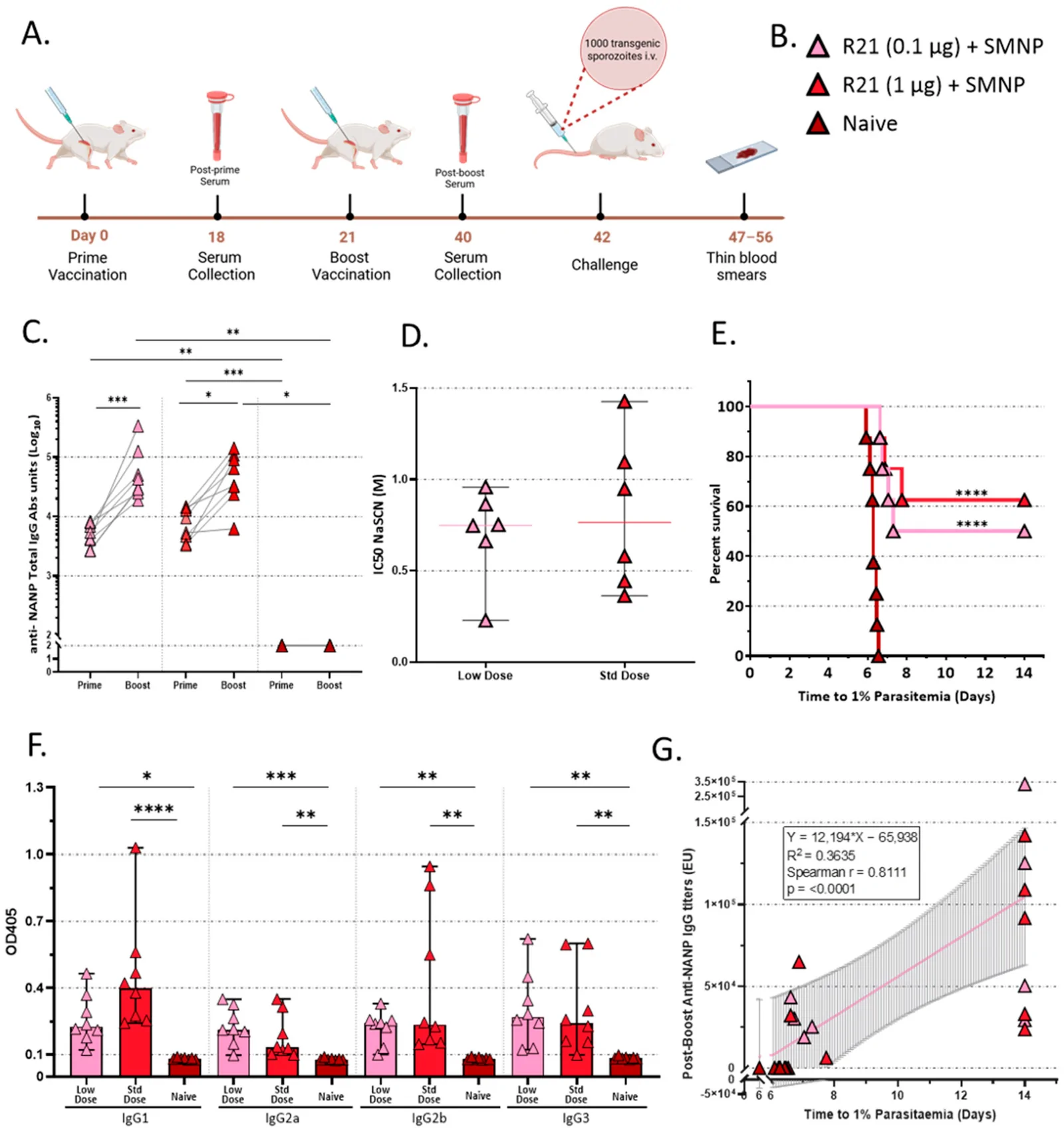
Low dose and standard dose of R21 formulated with SMNP provide moderate efficacy in *CD1* mice. (**A**) Experimental design. Here, 6-week-old female *CD1* mice (*n* = 8, *n* = 7 for naïve group) received two intramuscular immunizations, three weeks apart, containing either 0.1 µg or 1 µg R21 formulated with 5 µg of SMNP. Blood was collected via tail bleeding 2–3 days before boost vaccination and again before challenge. Approximately three weeks after boosting, mice were challenged intravenously with 1000 transgenic sporozoites. (**B**) Vaccination groups included in the study. (**C**) Anti-NANP IgG responses measured following prime and booster immunization. Samples yielding an OD below 0.1 were assigned a titer of 100 and classified as non-responders; these values are shown at the dotted threshold. Post-boost responses were compared with the naïve group using the Kruskal–Wallis test followed by Dunn’s multiple comparisons. (**D**) Avidity of NANP-specific IgG following booster immunization for the first six mice of each group. Data are shown as a median with a 95% confidence interval. Groups were compared using the Mann–Whitney test. (**E**) Protection following sporozoite challenge, shown as Kaplan–Meier curves. Differences between groups were evaluated using the log-rank (Mantel–Cox) test. (**F**) NANP-specific IgG subclass responses, shown as OD405 values for IgG1, IgG2a, IgG2b, and IgG3. For comparisons among independent groups, a Kruskal–Wallis test followed by Dunn’s multiple-comparisons test was used. Paired samples were analyzed using a Friedman test followed by Dunn’s multiple-comparisons test. (**G**) Relationship between post-boost anti-NANP IgG titers and time to 1% parasitemia. Data from all experimental groups were combined for this analysis. Mice remaining parasite-negative until the end of the study were assigned a value of 14 days. The fitted regression line and its 95% confidence interval are shown, together with the corresponding R^2^ value. Association between antibody titer and time to parasitemia was assessed using Spearman’s rank correlation. Statistical significance was defined as *p* ≤ 0.05 and displayed with asterisks (* = *p* ≤ 0.05, ** = *p* ≤ 0.01, *** = *p* ≤ 0.001, and **** = *p* ≤ 0.0001).

## Data Availability

The original contributions presented in this study are included in the article/Supplementary Material. Further inquiries can be directed to the corresponding authors.

## Supplementary Materials

The following supporting information can be downloaded at https://www.mdpi.com/article/10.3390/vaccines14100861/s1, Figure S1: Fold change in anti-NANP IgG titers in *BALB/c*, *C57BL/6*, and *CD1*; Figure S2: Relationship between post-boost avidity levels and time to 1% parasitemia in *BALB/c*, *C57BL/6*, *CD1*, and for all strains combined.
