# Supplementary material for: Preclinical Evaluation of the SMNP-Adjuvanted R21 Malaria Vaccine Across Multiple Mouse Strains
Source: Vaccines (Basel). 2026 Sep 28;14(10):861. doi: 10.3390/vaccines14100861 (PMC13709546; doi:10.3390/vaccines14100861)
Supplement: Supplementary file 1 [file vaccines-14-00861-s001.zip › vaccines-4540651-supplementary.pdf]

Supplementary Material

1. Supplementary Figures

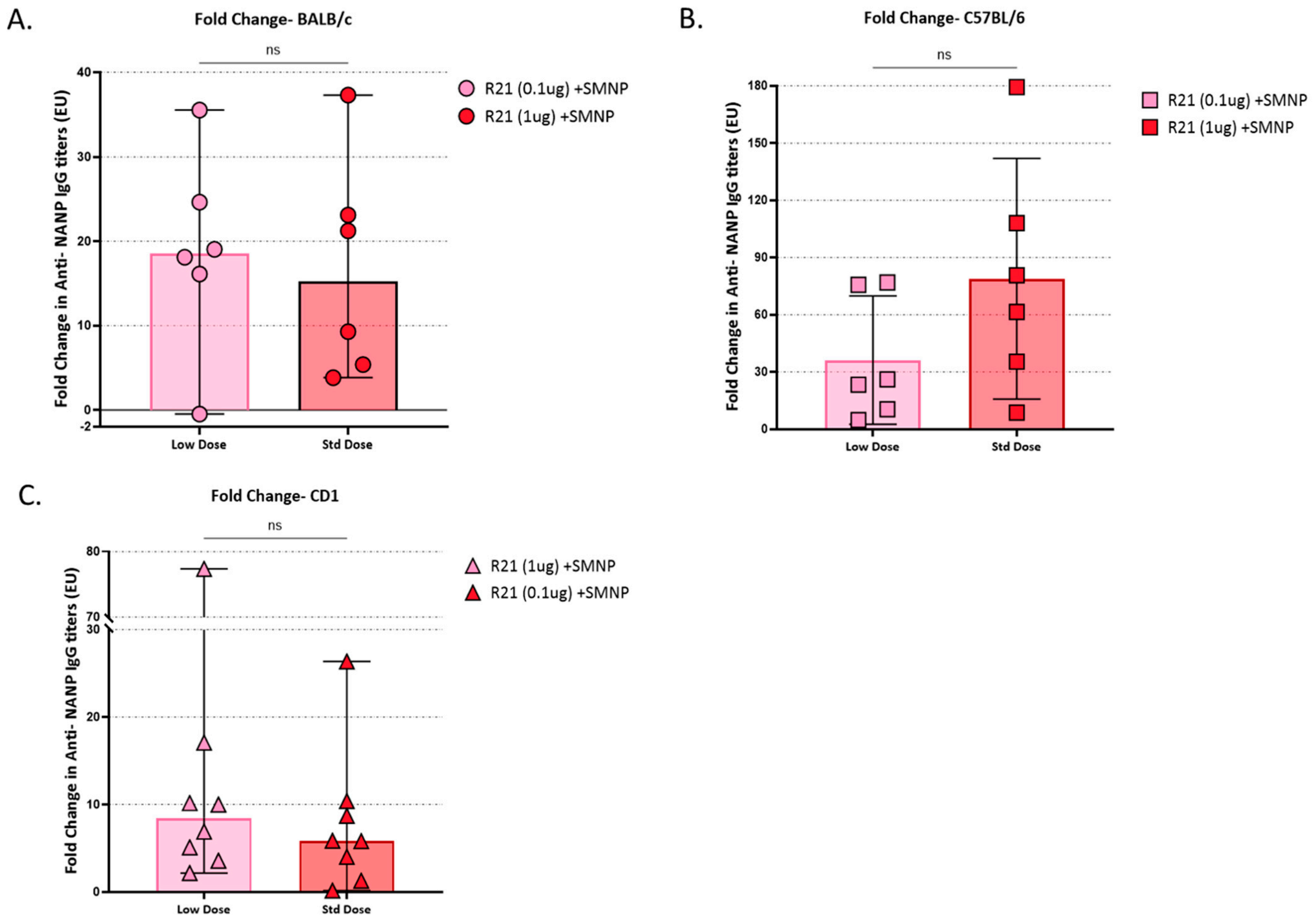

Supplementary Figure S1: Fold change in anti-NANP IgG titres in (A) BALB/c, (B) C57BL/6, and (C) CD1. Statistical analysis was performed using Mann-Whitney test. A value of p=0.05 or less was considered statistically significant for all analyses.

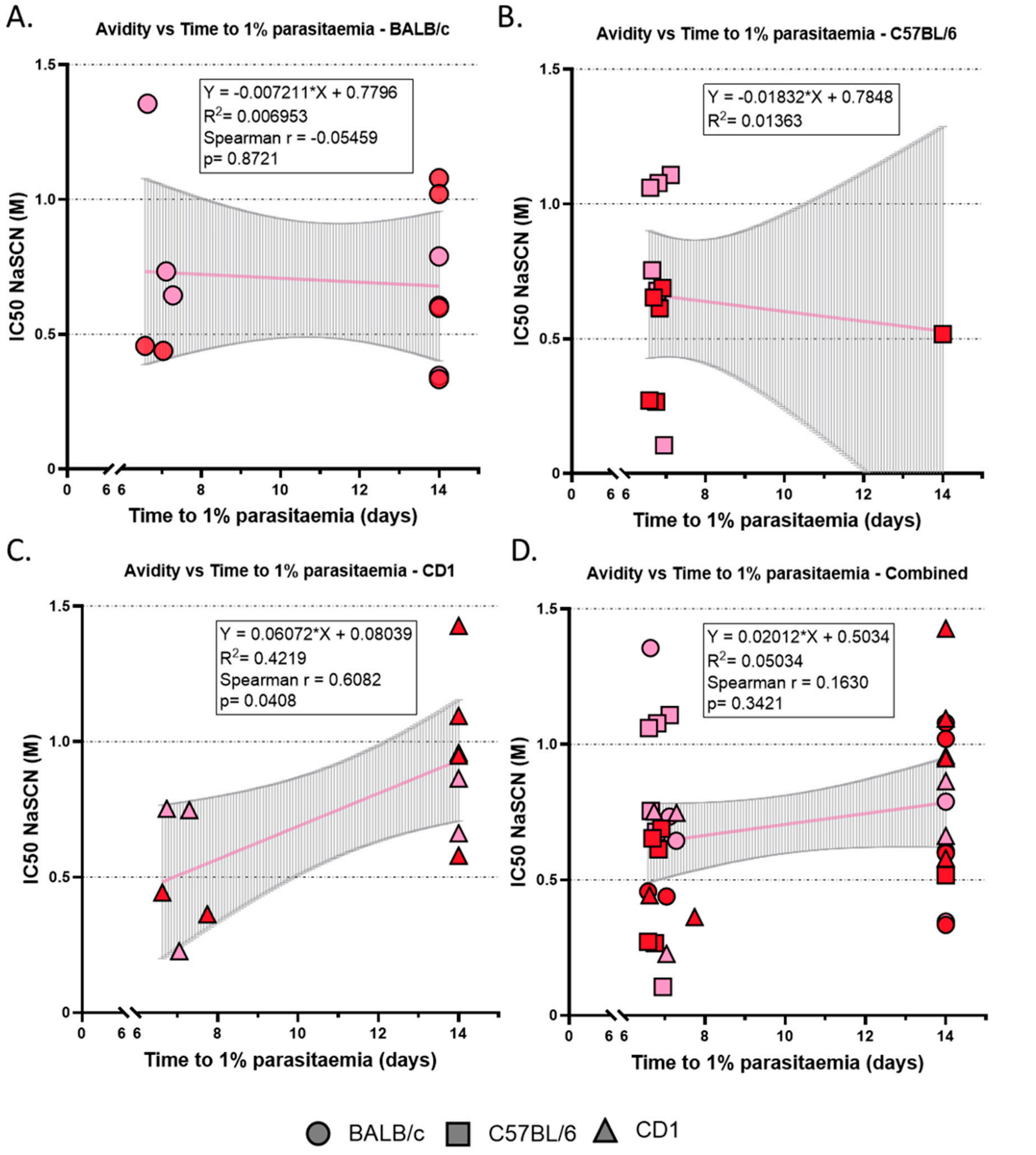

Supplementary Figure S2: Relationship between post-boost avidity levels and time to 1% parasitaemia in (A) BALB/c, (B) C57BL/6, (C) CD1, and (D) for all strains combined. Symbols indicate mouse strain: circles, BALB/c; squares, C57BL/6; triangles, CD1. Colours indicate vaccine dose: light pink, low-dose vaccine; red, standard-dose vaccine. Each point represents one mouse. Data from both vaccinated groups were combined for each analysis. Mice remaining parasite-negative until the end of the study were assigned a value of 14 days. The fitted regression line and its 95% confidence interval are shown, together with the corresponding R^2 value. Association between NaSCN molarity and time to parasitaemia was assessed using Spearman's rank correlation except for C57BL/6 only analysis as only 1 mouse was protected in this strain. Statistical significance was defined as $p \leq 0.05$ and displayed with asterisks (*=p≤ 0.05, **=p≤ 0.01, ***=p≤ 0.001, and ****=p≤ 0.0001).
